# Correlation between sentinel lymph node biopsy and non-sentinel lymph node metastasis in patients with cN0 breast carcinoma: comparison of invasive ductal carcinoma and invasive lobular carcinoma

**DOI:** 10.1186/s12957-024-03375-9

**Published:** 2024-04-17

**Authors:** Calogero Cipolla, Simona Lupo, Nello Grassi, Giuseppe Tutino, Martina Greco, D’Agati Eleonora, Vittorio Gebbia, Maria Rosaria Valerio

**Affiliations:** 1 Department of Surgical Oncological and Oral Sciences, University of Palermo, Palermo, Italy; 2 Breast Unit - AOUP Paolo Giaccone Palermo, Palermo, Italy; 3 UOC Medical Oncology - AOUP Paolo Giaccone Palermo, Palermo, Italy; 4 Medical Oncology, School of Medicine, University of Enna Kore, Enna, Italy; 5 Director Medical Oncology Unit, Cdc Torina, Palermo, Italy; 6 Co-coordinator scientific research, Humanitas Istituto Clinico Catanese, Misterbianco, Catania Italy

**Keywords:** Breast carcinoma, Infiltrating lobular carcinoma, Infiltrating ductal carcinoma, Sentinel lymph node biopsy, Non-sentinel lymph node metastasis, Axillary lymphadenectomy

## Abstract

Some studies have suggested that axillary lymph node dissection (ALND) can be avoided in women with cN0 breast cancer with 1–2 positive sentinel nodes (SLNs). However, these studies included only a few patients with invasive lobular carcinoma (ILC), so the validity of omitting ALDN in these patients remains controversial. This study compared the frequency of non-sentinel lymph nodes (non-SLNs) metastases in ILC and invasive ductal carcinoma (IDC). Materials Methods: Data relating to a total of 2583 patients with infiltrating breast carcinoma operated at our institution between 2012 and 2023 were retrospectively analyzed: 2242 (86.8%) with IDC and 341 (13.2%) with ILC. We compared the incidence of metastasis to SLNs and non-SLNs between the ILC and IDC cohorts and examined factors that influenced non-SLNs metastasis. Results: SLN biopsies were performed in 315 patients with ILC and 2018 patients with IDC. Metastases to the SLNs were found in 78/315 (24.8%) patients with ILC and in 460 (22.8%) patients with IDC (*p* = 0.31). The incidence of metastases to non-SLNs was significantly higher (*p* = 0.02) in ILC (52/78–66.7%) compared to IDC (207/460 − 45%). Multivariate analysis showed that ILC was the most influential predictive factor in predicting the presence of metastasis to non-SLNs. Conclusions: ILC cases have more non-SLNs metastases than IDC cases in SLN-positive patients. The ILC is essential for predicting non-SLN positivity in macro-metastases in the SLN. The option of omitting ALND in patients with ILC with 1–2 positive SLNs still requires further investigation.

## Introduction

Sentinel lymph node biopsy (SLNB) has now been confirmed as a standard surgical procedure for staging the axilla in patients with early breast cancer (BC) and clinically negative lymph nodes (cN0), limiting the use of axillary lymph node dissection (ALND) to patients with positive SLNs [1, 2]. However, positivity in non-SLNs is only found in approximately 34–50% of patients with positive SLNs undergoing completion of ALND [3]. The ACOSOG Z0011 study demonstrated that omitting ALND in cT1-2 cN0 cM0 patients with 1–2 positive SLNs resulted in a non-inferior outcome compared to patients undergoing ALND [4]. However, patients with invasive ductal carcinoma (IDC) constitute more than 80% of the ACOSOG Z0011 study population. For this reason, some questions have been raised about applying its findings to other histological types, particularly invasive lobular carcinoma (ILC).

Although ILC represents only approximately 5–10% of all BC, its immunophenotypic characteristics, clinical course, and therapy response present unique aspects that require particular attention. ILC more frequently shows positivity for hormone receptors and little or no expression for human epidermal growth factor receptor-2 (HER2) compared to IDC. The response to chemotherapy is significantly lower than that of IDC [5–8].

Recent studies have shown that ILC has a similar rate of metastasis to non-SLNs compared to IDC, thus supporting the idea that applying the ACOSOG Z0011 criteria is safe even in patients with ILC [9, 10]. However, information regarding the implementation of SLNB in ​​patients with ILC is still scarce, and the question remains unclear whether patients with ILC and 1–2 positive SLNs can be exempted from ALND [10–12] without effects on recurrences and survival.

In this study, we retrospectively compared the rates of metastatic lymph node involvement in non-SLNs between patients with IDC and those with ILC, intending to offer a further contribution to the question of whether the ACOSOG Z0011 trial criteria can also be safely applied to patients with ILC.

## Materials and methods

### Study design

After approval by the Institutional Review Board of the University Hospital AUOP Paolo Giaccone of Palermo, we collected and retrospectively analyzed the clinical records of an extensive series of patients with cN0 primary invasive BC observed at our institution between 2012 and 2023. The aim of this retrospective study was to compare the rates of metastatic lymph node involvement in non-SLNs between patients with IDC and those with ILC to confirm of the ACOSOG Z0011 trial criteria can also be safely applied to patients with ILC.

### Inclusion and exclusion criteria

Data relating to patients with IDC and ILC who underwent SLNB were included in the study.

Exclusion criteria from the study were previous neoadjuvant therapy, inflammatory BC, locally recurrent BC, metastatic disease at the diagnosis, and lack of complete data. In all cases, the diagnosis of BC was made using a percutaneous biopsy with a 14G tru-cut needle or a vacuum-assisted breast biopsy with a 7G cannula [13]. Histopathological diagnoses of ILC and IDC were made with hematoxylin-eosin staining. Furthermore, the expressivity of the hormone receptors for estrogen and progesterone and HER-2 and the Ki-67 cell proliferation index were evaluated. According to the St. Gallen’s 2013 consensus conference, tumors were then classified based on molecular subtypes. The diagnosis of IDC was confirmed by positive immunohistochemical staining for E-cadherin. Clinical evaluation of the axilla was performed for all patients with clinical examination, ultrasound, and cytological examination using FNAC of the suspicious lymph nodes. All patients with positive FNA were considered cN+, even those in whom the lymph nodes were not palpable. Women with clinically negative axillary lymph nodes underwent SLNB. The SLN was detected using the radiotracer identification technique and, if necessary, using vital dye, as described in our previous studies [14–16]. All patients underwent synchronous breast cancer excision by breast-conserving surgery or total mastectomy and SLNB. The recovered SLNs were analyzed during surgery using frozen section (FS) histological examination. All SLNs were subsequently examined with definitive histopathological examination complete with immunohistochemistry [16]. To reduce the false negative rate, in cases where suspicious nodes were present on intraoperative palpation of the axilla, these were removed and sent to the FS for histological examination along with SLNs as they were considered as such. Completion ALND was not performed in patients with negative SLNs, isolated tumor cells (ITC), or micrometastases. In the case of macrometastases at FS in the SLN or in any suspicious nodes removed simultaneously, the patients immediately underwent completion ALND. In cases of SLN or palpable suspicious nodes negative at FS but positive for macrometastases at the definitive histopathological examination, ALND was completed in a second operation.

Whole breast radiotherapy was done in all patients underwent breast conservative surgery. Patients with > 3 positive axillary lymph nodes underwent radiotherapy of the lymph glandular stations.

### Statistical analysis

Due to the retrospective chart review and the binomial primary endpoint, we applied a statistical power analysis to determine the appropriate sample size. Being 66.7% (p1), 45% (p2) the incidence of groups 1 and 2, Δ = 21.7 (p2-p1) the absolute difference between two proportions, 78 (n1) the sample size for group 1, 460 (n2) the sample size for group 2, the probability of type I error alpha of 0.05, z = critical Z value for a given α or β, K ratio of sample size for group 2 to group 1, and the Φ function converting a critical Z value to power, the post-hoc power of the study was 95.2%.

Differences between the two patient cohorts were calculated using the χ2 test. The statistical significance limit was defined as a *p*-value < 0.05. A logistic regression analysis was performed to examine the factors that influenced the presence of metastases to non-SLNs when the SLN had macro-metastases.

## Results

The clinical records of 2583 patients with infiltrating BC operated between 2012 and 2023 were evaluated: 2242 (86.8%) with IDC and 341 (13.2%) with ILC. Two hundred twenty-four patients with IDC and 26 patients with ILC were excluded from the study as they initially underwent ALND. Ultimately, 2333 patients were included in the study, of which 2018 (86.4%) had IDC and 315 (13.5%) had ILC.

### Patients’ population

The clinical and pathological characteristics of the two cohorts of patients are summarized in Table 1. Patients with ILC were older at diagnosis than those with IDC (*p* < 0.01). The Luminal A molecular subtype was the most represented in ILC compared to IDC, unlike the HER2-enriched and Triple Negative subtypes, which were lower in ILC than DCI (*p* < 0.001). Furthermore, ILCs were found to have a larger diameter (*p* < 0.01) and a higher histological grade (*p* < 0.01) compared to IDCs.

**Table 1:** Clinical and pathological characteristics of patients

Characteristic	IDC (n. 2018 pts)	ILC (n. 315 pts)	*P* value
	N (%)	N (%)	
Age at diagnosis			
< 50	207 (10.3)	8 (2.5)	< 0.01
≥ 50	1811 (89.7)	307 (97.5)	
Pathological tumor size			
T1	1271 (62.9)	201 (63.8)	
T2	689 (34.1)	92 (29.2)	< 0.01
T3	58 (2.9)	22 (6.9)	
Tumor Grade			
G1	365 (18.1)	43 (13.7)	
G2	1199 (59.4)	211 (66.9)	< 0.01
G3	454 (22.5)	61 (19.4)	
Molecular subtype			
Luminal A	594 (29.4)	132 (41.9)	
Luminal B	917 (45.4)	152 (48.2)	< 0.001
HER2 enriched	263 (13.1)	13 (4.1)	
TNBC	244 (12.1)	18 (5.7)	
Surgical treatment			
Breast conservative surgery	1427 (70,7)	218 (69.2)	0.89
Total Mastectomy	591 (29.3)	97 (30.8)	
Number of resected SLNs			
Average number	3.9	4.2	0.72
Range	5-Jan	1–7	

The presence of metastases in the SLN was found in 93 patients (29.5%) with ILC; 15 cases were excluded as they were micrometastases, and in the end, a macrometastases was found in 78 of the 315 patients (24.8%) with ILC. The presence of metastases in the SLN was found in 607 patients (30%) with IDC; 147 cases were excluded as they were micrometastases, and ultimately, a macrometastases was found in 460 of the 2018 patients (22.8%) with ILC. The difference between the two groups was not significant (p 0.31).

**Fig. 1:**
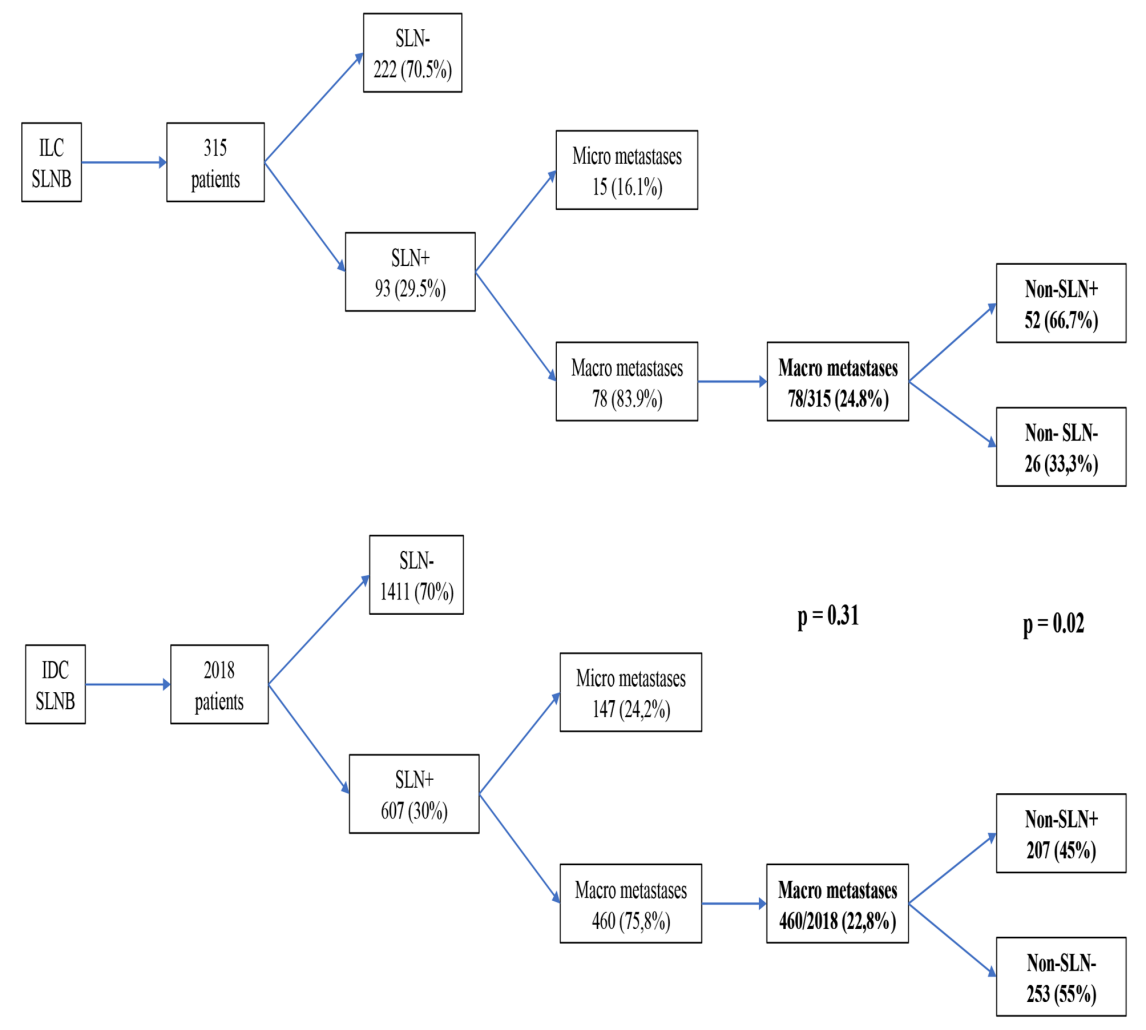
Study flow-chart

Metastases to non-SLNs were found in 52/78 patients (66.7%) with ILC and in 207/460 patients (45%) with positive SLNs and undergoing completion ALND, with a statistically significant difference between the two groups (*p* = 0.02). The data are summarized in Fig. 1. Furthermore, as can be seen in Table 2, the number of metastatic non-SLNs was significantly higher in ILC compared to IDC.

**Table 2:** Number of metastatic non-SLNs, comparison between ILC and IDC

Number of metastatic non-SLN	IDC 207 SLN + patients N (%)	ILC 78 SLN + patients N (%)
0	253 (55)	26 (33.3%)
1	97 (21.1)	16 (20.5)
2	31 (6.7)	6 (7.7)
3	18 (3.9)	9 (11.5)
4 or more	61 (13.2)	21 (26.9)

The multivariate analysis also showed that in patients with macrometastases in the SLN, in addition to the number of positive SLNs, the ILC histotype represents the most influential factor in predicting the presence of metastases in non-SLNs concerning age, tumor size, grade histological and molecular subtype (Table 3).

**Table 3:** Multivariate analysis for non-SLN metastases

Variables	OR	95% CI	*P* value
Pathology (ILC/IDC)	2.81	1.09–7.41	0.037
Age (≥ 50/<50)	0.61	0.32–1.19	0.142
Tumor size			
T1	0.87	0.17–4.91	0.861
T2	1.74	0.27–11.01	0.521
T3	1.16	0.14–9.32	0.919
Tumor Grade			
G1	1.00		
G2	1.19	0.65–2.19	0.589
G3	0.90	0.43–1.88	0.756
Molecular subtype			
Luminal A	1.47	0.77–2.79	0.232
Luminal B	1.22	0.98–2.42	0.751
HER2 enriched	1.74	0.93–2.94	0.813
TNBC	1.78	0.82–2.99	0.643
Number of positive SLN (≥ 3 / ≤2)	4.97	1.65-1	0.005

## Discussion

In our study, patients with ILC had a higher mean age, larger tumor size, and higher grading than patients with IDC. Furthermore, luminal molecular subtypes were more represented in ILCs than IDCs, in contrast to HER2-enriched and Triple Negative subtypes, which were lower in ILCs than DCIs. These data align with those reported in the literature on the characteristics of the ILC [17–19]. Regarding the number of SLNs removed and the number of metastatic SLNs, although higher in ILC, no significant difference was found between the two groups of patients. This is also consistent with the results of previous studies [17].

However, among patients with macro-metastases in the SLN, those with ILC more frequently had metastases to non-SLNs than patients with IDC (66.7% ILC vs. 45% IDC *p* = 0.02). The multivariate analysis highlighted that ILC is the most influential factor in predicting the presence of metastases to non-SLNs in patients with macro-metastases in the SLN. This data is comparable to that reported in previous studies, which have demonstrated that the ILC tends to have a more significant number of positive non-sentinel lymph nodes [10, 20–23]. However, the issue remains controversial, given that other authors have reported opposite results, concluding that ILC histology is not associated with a greater risk of metastatic involvement of non-sentinel axillary lymph nodes [11, 24].

The reason for a greater number of metastatic axillary lymph nodes in the ILC may lie in the loss of function of E-cadherin, a trans-membrane protein, typically absent in the ILC, which forms bonds in the extracellular space that joins the plasma membrane to actin and the microtubule cytoskeleton. Its loss would decrease cohesion between tumor cells, increasing the rate of multiple metastases [20, 22]. Furthermore, previous studies have demonstrated that ILC infiltration typically lacks desmoplastic reaction and does not destroy anatomical structures. Consequently, nodal metastases in the ILC may be more challenging to detect at diagnosis through imaging and may increase the number of metastases to non-SLNs [22, 25]. Furthermore, in some studies, it is reported that the false negative rate of ultrasound-guided fine needle biopsies was higher in ILC than in IDC because small, uniform cells without nuclear atypia are found in ILC, and the distinction between tumor cells and histiocytes is difficult. This issue may also underlie the underestimation of nodal status at diagnosis [20, 26].

Other studies have identified the size of SLNs metastases and extracapsular invasion into the SLN as predictive factors for non-SLNs positivity after SLNB. They also demonstrated that patients with micro-metastases in the SLN have a lower incidence of metastasis to non-SLNs than those with macro-metastases. However, these predictive factors have been determined in patients affected predominantly by IDC and a small percentage of ILC [3, 20, 21, 25, 27, 28]. For these reasons, further studies are undoubtedly needed to examine the predictive factors of non-sentinel lymph node positivity in cohorts of ILC patients with macro-metastases in the SLN.

In the ASOCOG Z0011 trial, patients randomized to SLNB followed by ALND had a non-SLN positivity rate of 27% [4]. Roberts et al. analyzed the treatment of the axilla in ILC, reporting a positivity of 40% for non-SLNs. However, when ILCs meet the ACOSOG Z001 criteria, non-sentinel lymph node positivity dropped to 17% [9]. Gao et al. found that ILC had similar rates of metastasis to non-SLNs compared to IDC among patients with 1–2 positive SLNs (31.2% in ILC vs. 28.6% in IDC, *p* = 0.481) [11]. However, the study comprised 182 patients with IDC and only 5 patients with ILCs and 1–2 SLNs positive. In contrast, in the AMAROS trial, ILC cases had a rate of metastasis to non-SLNs of 43%, higher than that of all other tumor types [29]. Zhang et al. reported a higher incidence of metastasis in non-SLNs for ILC compared to IDC among patients with 1–2 positive SLNs. However, the difference was not significant (45.4% in ILC, *n* = 30 vs. 34, 8% in IDC, *n* = 1,122, *P* = 0.366) [30]. Therefore, surgeons should be more cautious in omitting ALND for ILC patients with 1–2 positive SLNs.

It should also be considered that omission of ALND in patients with positive SLNs who meet ASOCOG criteria may result in underdiagnosis of axillary lymph nodes. This could lead to undertreatment of those patients with ≥ 4 positive lymph nodes who could instead benefit from adjuvant treatment with CDK4/6 inhibitors which however is available in Italy since July 2023 only. The MONARCHE trial showed that abemaciclib, in combination with hormone therapy, demonstrated a significant improvement in disease-free survival in patients with hormone-positive, HER2-negative, node-positive early breast cancer at high risk of early recurrence [31].

Consequently, there is a need for more reliable data in the literature on the prognosis of ILC after the omission of ALND. Some studies have shown that overall survival was significantly higher in ILC than in IDC [6, 17]. However, numerous other studies have shown that ILC, despite favorable biological characteristics, does not have a better clinical outcome than IDC [10, 17, 18].

Furthermore, ILC appears to have a lower response to chemotherapy than IDC [7, 10]. Although some studies have suggested that radiotherapy after conservative surgery has the same loco-regional control in both ILC and IDC [32], no study has demonstrated the difference in sensitivity to radiotherapy between ILC and IDC. Therefore, it would be appropriate to include the sensitivity of the ILC to adjuvant therapies in the decision-making process for omitting ALND in patients with 1–2 positive SLNs.

Although it was conducted on a large series of patients, our study has some limitations as it is retrospective, includes data from only one institution, and the number of patients with ILC is relatively low compared to that of patients with IDC.

## Conclusions

However, we would like to conclude that the ILC presents more metastases to non-SLNs than the IDC and that it must be considered an important predictive factor for the positivity of non-SLNs in cases of macro-metastasis to the SLNs. Consequently, omitting ALND in patients with ILC who meet the ASOCOG Z0011 trial criteria may underestimate the number of metastatic axillary lymph nodes, risking less accurate staging and selecting less effective adjuvant therapy. The decision to omit ALND in ILC with positive sentinel lymph nodes requires a more thorough evaluation.

## Data Availability

The data analyzed during the current study are available from the corresponding author on reasonable request.
